# Increased interhemispheric somatomotor functional connectivity and mirror overflow in ADHD

**DOI:** 10.1016/j.nicl.2021.102759

**Published:** 2021-07-10

**Authors:** C. Chen, D. Lidstone, D. Crocetti, S.H. Mostofsky, M.B. Nebel

**Affiliations:** a Center for Neurodevelopmental and Imaging Research, Kennedy Krieger Institute, 716 North Broadway, Baltimore, MD 21205, USA; b Department of Neurology, Johns Hopkins University, 733 North Broadway, Baltimore, MD 21205, USA; c Department of Psychiatry and Behavioral Sciences, Johns Hopkins University, 733 North Broadway, Baltimore, MD 21205, USA

**Keywords:** Interhemispheric functional connectivity, fMRI, Motor control, Motor cortex, ADHD

## Abstract

Mirror overflow is a developmental phenomenon defined as unintentional movements that mimic the execution of intentional movements in homologous muscles on the opposite side of the body. In children with attention-deficit/hyperactivity disorder (ADHD), mirror overflow is commonly excessive, abnormally persistent, and correlated with ADHD symptom severity. As such, it represents a promising clinical biomarker for disinhibited behavior associated with ADHD. Yet, the neural underpinnings of mirror overflow in ADHD remain unclear. Our objective was to test whether intrinsic interhemispheric functional connectivity between homologous regions of the somatomotor network (SMN) is associated with mirror overflow in school age children with and without ADHD using resting state functional magnetic resonance imaging. To this end, we quantified mirror overflow in 119 children (8–12 years old, 62 ADHD) during a finger sequencing task using finger twitch transducers affixed to the index and ring fingers. Group ICA was used to identify right- and left-lateralized SMNs and subject-specific back reconstructed timecourses were correlated to obtain a measure of SMN interhemispheric connectivity. We found that children with ADHD showed increased mirror overflow (*p* < 0.001; *d* = 0.671) and interhemispheric SMN functional connectivity (*p* = 0.023; *d* = 0.521) as compared to typically developing children. In children with ADHD, but not the typically developing children, there was a significant relationship between interhemispheric SMN functional connectivity and mirror overflow (*t* = 2.116; *p* = 0.039). Our findings of stronger interhemispheric functional connectivity between homologous somatomotor regions in children with ADHD is consistent with previous transcranial magnetic stimulation and diffusion-tractography imaging studies suggesting that interhemispheric cortical inhibitory mechanisms may be compromised in children with ADHD. The observed brain-behavior correlation further suggests that abnormally strong interhemispheric SMN connectivity in children with ADHD may diminish their ability to suppress overflow movements.

## Highlights

• Resting state fMRI study of the neurobiological basis of mirror overflow in ADHD.
• Children with ADHD show greater interhemispheric motor functional connectivity.
• Children with ADHD show greater mirror overflow during finger sequencing.
• Interhemispheric functional connectivity correlates with mirror overflow in ADHD.
• Greater interhemispheric motor connectivity may impede overflow inhibition in ADHD.

## 1 Introduction

Attention deficit/hyperactivity disorder (ADHD) is a neurodevelopmental disorder characterized by inattention, hyperactivity, and impulsiveness (Danielson et al., 2018). Children with ADHD also tend to demonstrate excessive and abnormally persistent motor overflow. In particular, they demonstrate mirror overflow, a subtype of motor overflow defined as a developmental phenomenon defined as unintentional movements that mimic the execution of intentional movements in homologous muscles on the opposite side of the body (Mostofsky et al., 2003, Cole et al., 2008, MacNeil et al., 2011, Gaddis et al., 2015, McAuliffe et al., 2019). Mirror overflow is common in early childhood and typically decreases as children age into adolescence. However, both qualitative (Physical and Neurological Examination for Subtle Signs; (Denckla, 1985)) and quantitative (finger twitch transducers; Biopac Systems Inc., Goleta, CA) assessments suggest that mirror overflow is greater and more likely to persist longer in children with ADHD, particularly boys with ADHD, compared to typically developing (TD) children (MacNeil et al., 2011, Mostofsky et al., 2003, Cole et al., 2008; (Denckla and Rudel, 1978)). Given the developmental nature of ADHD, brain-based mechanisms critical for inhibiting unwanted movements may contribute to impaired inhibitory capacity more generally in children with ADHD; yet, the neurobiological basis underlying excessive motor overflow in ADHD remains unclear.

Transcranial magnetic stimulation (TMS) and diffusion-tensor imaging (DTI) studies suggest interhemispheric cortical inhibitory mechanisms are compromised in children with ADHD (Wu et al., 2012, Wu et al., 2017). Additionally, functional magnetic resonance imaging (fMRI) and electroencephalogram (EEG) studies have shown that abnormal recruitment of primary motor cortex (M1) contralateral to intentional movement in children with ADHD is associated with increased mirror overflow (Gaddis et al., 2015, McAuliffe et al., 2019). The observed reduced recruitment of contralateral M1 in these studies may reflect reduced activation of inhibitory connections on the homologous M1, which, in turn, is behaviorally expressed as mirror overflow. However, to date, no research has directly examined interhemispheric functional connectivity within the motor control system.

We aimed to test whether intrinsic functional connectivity between homologous left and right somatomotor regions was associated with increased mirror overflow. Given that greater functional connectivity between brain regions reflects reduced functional segregation, we hypothesized that children with ADHD would display significantly greater interhemispheric functional connectivity between homologous somatomotor regions compared to TD children, and that, among children with ADHD, increased SMN interhemispheric functional connectivity would be associated with increased mirror overflow.

## 2 Methods

### 2.1 Standard protocol approvals, registrations, and patient consents

These studies were approved by the Johns Hopkins University Institutional Review Board. A parent or guardian of the participants signed informed consent and all participants provided verbal assent.

### 2.2 Participants and diagnostic criteria

One hundred and nineteen 8- to 12-year-old children were included in this sample: 62 children with ADHD (mean age = 10.4 years, standard deviation = 1.4 years) and 57 TD children (mean age = 10.6 years, standard deviation = 1.2 years) (Table 1). These participants were drawn from a larger sample of children who participated in one of several studies of ADHD at our center between 2008 and 2019, including 96 children with ADHD (69 boys) and 65 TD children (43 boys). Analyses focused on a subset of this sample after excluding participants who moved excessively during the rsfMRI scan (n = 46, 34 ADHD). Participants were principally recruited through local public schools, but also through community-wide advertisement, volunteer organizations, medical institutions, daycare centers, websites, and word of mouth. Participants were screened via parent telephone interview to determine eligibility.

**Table 1:** Demographic and behavioral information by group.

		Typically developing (*n* = 57)	ADHD (*n* = 62)	
Sex (M/F)		36/21	45/17	*p* = 0.27
Age (Years)	Behavioral age	10.6 ± 1.2	10.4 ± 1.4	*p* = 0.39
	Scan age	10.7 ± 1.2	10.5 ± 1.4	*p* = 0.44
Handedness	Right/Mixed	54/2	59/1	*p* = 0.71
	Edinburgh	0.82 ± 0.17	0.88 ± 0.15	*p* = 0.07
FSIQ		114.8 ± 11.4	106.3 ± 13.5	***p* < 0.001**
SES		55.4 ± 10.2	51.8 ± 11.2	*p* = 0.08
Mean FD (mm)		0.20 ± 0.12	0.28 ± 0.16	***p* = 0.002**
Conners (CBRS)	Inattentive	45.0 ± 5.5	73.4 ± 10.8	***p* < 0.0001**
	Hyperactive	46.7 ± 5.1	72.5 ± 12.3	***p* < 0.0001**
Total mirror overflow		382.0 ± 303.1	690.3 ± 574.4	***p* < 0.001**
(min *-* max)		(41.5–1441.7)	(69.9–2807.0)	

Children with histories of neurologic illness or injury, genetic disorders, seizures, or intellectual disability were excluded from the study. Children with a full scale IQ (FSIQ) <80 on the Wechsler Intelligence Scale for Children, Fourth or Fifth Edition (WISC-IV or WISC-V; Wechsler, 2003, Wechsler, 2014) were excluded.

### 2.3 ADHD diagnosis

A diagnosis of ADHD was determined using the DSM-IV or DSM–5 (American Psychiatric Association, 2000, American Psychiatric Association, 2013) criteria and confirmed using a structured parent interview, either the Diagnostic Interview for Children and Adolescents-IV (Reich et al., 1997) or the Kiddie Schedule for Affective Disorders and Schizophrenia for School-Age Children (K-SADS; Kaufman et al., 2016), as well as parent and teachers versions of either the Conners-Revised (n = 55) or Conners-3 (n = 62) Rating Scale (Conners, 1997, Conners, 2008). Diagnosis was verified by a board-certified child neurologist (SHM) or licensed clinical psychologists all with extensive experience in the clinical assessment of children with ADHD and related disorders.

Children with ADHD were excluded from the study if they met criteria, based on the Diagnostic Interview for Children and Adolescents-IV or the Kiddie Schedule for Affective Disorders and Schizophrenia for School-Age Children, for active psychosis, major depression, bipolar disorder, conduct disorder, adjustment disorder, or anxiety disorders, including generalized anxiety disorder, separation anxiety disorder, and social phobias, and obsessive–compulsive disorder. Children with ADHD were allowed to have comorbid oppositional defiant disorder (n = 20).

Children taking non-stimulant medications, selective serotonin reuptake inhibitors, or other psychotropic medications were excluded. Those taking stimulant medications (34%) were asked to withhold their medications the day of and the day prior to participating in the study to avoid effects of stimulants on cognitive, behavioral, and motor measures.

Children were included in the TD group only if they did not meet diagnostic criteria on either the diagnostic interview or the ADHD rating scale. Children in the TD group were deemed ineligible if they were taking any psychotropic medications, such as stimulants or mood stabilizers.

### 2.4 fMRI acquisition and preprocessing

All participants completed a mock scanning session to habituate to the MRI environment. Resting state fMRI (rsfMRI) scans were acquired on a Philips 3 T scanner using an 8-channel (n = 35, 156 time points; n = 23, 128 time points) or a 32-channel (n = 61, 156 time points) head coil using a single-shot, partially parallel, gradient-recalled echo planar sequence with sensitivity encoding (repetition time [TR]/echo time = 2500/30 ms, flip angle = 70°, sensitivity encoding acceleration factor of 2, 3-mm axial slices with no slice gap, in-plane resolution of 3.05 X 3.15 mm [84 X 81 acquisition matrix]). An ascending slice order was used, and the first 10 s were discarded at the time of acquisition to allow for magnetization stabilization.

Functional MRI preprocessing procedures for group independent components analysis (group ICA) are described in detail elsewhere (Nebel et al., 2016). For quality control, no data contained between-volume translational movements larger than 3 mm or rotational movements larger than 3°. Volumes at the beginning and/or end of the rsfMRI scans were removed if needed to enforce this criterion, but at least 5 min of data were retained from each participant; as explained above, this resulted in the exclusion of 46 children (34 with ADHD).

### 2.5 Independent component analysis

Group spatial ICA was used to identify temporally coherent brain networks in children with ADHD and TD controls (GIFT v4.0b: http://icatb.source forge.net/; Medical Image Analysis Lab, Albuquerque, New Mexico). Prior to running group ICA, the dimension of each scan was estimated using an information theoretic approach (Li et al., 2007), and the maximum dimension estimate across participants was selected as the number of independent components (ICs) for the entire group (model order = 56). Using this moderately large model order ensured that the group decomposition would include separate right and left somatomotor components (Abou-Elseoud et al., 2010). Achieving this level of lateralization within the components estimated to represent the somatomotor network was necessary for addressing our hypotheses. This higher order ICA also allowed for better separation of signal and noise components.

Prior to ICA, each participant’s preprocessed data were reduced to 112 principle components (PCs) using principal component analysis to decrease computational demands, denoise the fMRI signal, and improve back-reconstruction of participant-specific spatial maps (SMs) and component timecourses (TCs) (Erhardt et al., 2011). Participant-specific PCs were temporally concatenated, and a second group principal component analysis reduced the data to 56 PCs using multi-power iteration (Rachakonda et al., 2016).

ICA was repeated on the 56 group-level PCs 100 times using the Infomax algorithm (Bell and Sejnowski, 1995) with randomized initial conditions as implemented in the Group ICA of fMRI Toolbox (GIFT) to ensure stability of the decomposition (Himberg, Hyvärinen, & Esposito, 2004). ICs were clustered across iterations, modes of each cluster were identified, and aggregate SMs were taken from the iteration with the maximum number of modes (Himberg et al., 2004). Subject-specific SMs and TCs were generated from aggregate ICs using a method based on principal component analysis compression and projection (Calhoun, Adali, Pearlson, & Pekar, 2001).

### 2.6 SMN functional connectivity

**Fig. 1:**
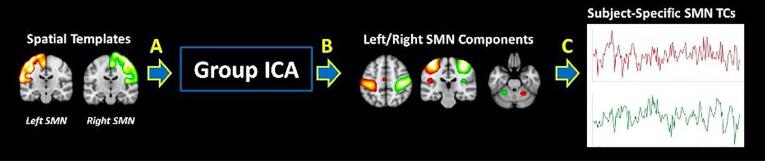
Definition of spatial maps of the somatomotor network.

We were specifically interested in extracting two resting-state somatomotor networks spatially corresponding to the left and right pre- and post-central gyri (see Fig. 1). Spatial maps of the right and left pre- and post-central gyri were generated using the Eve Atlas and smoothed with a Gaussian kernel to match the smoothness of the components (Mori et al., 2008, Nebel et al., 2016). Group-level components with the highest correlation to the SMN maps were extracted. Two-sample *t*-tests confirmed that the spatial topography of subject-specific left and right SMN SMs were not significantly different between groups.

Prior to calculating interhemispheric functional connectivity, left and right SMN TCs were detrended and despiked using 3dDespike (AFNI: http://afni.nimh.nih.gov/afni) as an additional precaution against lingering noise artifact (Allen et al., 2011). Pearson correlations between left- and right-lateralized participant-specific SMN TCs were computed and Fisher’s z transformation was applied to normalize the distribution of correlations. Following this transformation, the boundaries of possible values of SMN functional connectivity range from negative infinity to positive infinity.

### 2.7 Finger twitch transducers

**Fig. 2:**
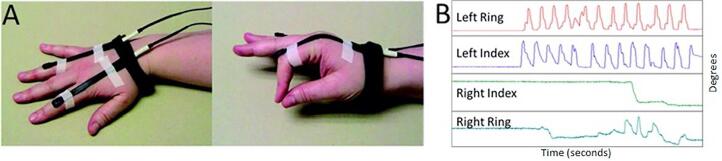
Administration and data collection of finger sequencing task.

Finger twitch transducers (Part #: TSD131-MRI; Biopac Systems Inc., Goleta, CA) were used to quantify mirror overflow (i.e., unintentional movements that mimic the execution of intentional movements on the opposite side of the body) in degrees of displacement from a baseline position. Transducers were affixed to the index and ring fingers of the left and right hands over the metacorpophalangeal (MCP) joint to capture extension and flexion (see Fig. 2**A**). The transducers were calibrated at 0° and 45° using AcqKnowledge software 4.2v (Biopac Systems Inc.) prior to beginning the task.

### 2.8 Finger sequencing task

Participants were asked to complete a sequential finger tapping task (outside of the scanner) to assess mirror overflow in the non-tapping hand (in degrees of displacement) (see Fig. 2**B**). Starting hand (left hand sequencing or right hand sequencing) for the first block was counterbalanced within diagnosis and sex. Ten blocks (five per hand, alternating hands) of finger sequencing were collected. Correct positioning showed the tapping hand positioned upright with the palm of the hand facing the camera in front of the participant and the non-tapping hand resting over a pillow on the participant’s lap to avoid restriction of extension and flexion in their fingers, thus allowing for overflow movements. Ten seconds of baseline were collected before each block during which the tapping hand was held in tapping position, as described above, without tapping. Participants were instructed to tap the pads of their fingers to their thumb in sequence (one sequence: index-middle-ring-pinky) “as big and fast” as possible to ensure valid, independent taps. 11 sequences (45 taps) were collected per block. To calculate a total overflow (TOF) score, we summed overflow in the non-tapping hand across all left- hand finger sequencing and right-hand finger sequencing blocks (MacNeil et al., 2011). With this method of calculation, the boundaries of possible values of TOF range from zero to positive infinity.

### 2.9 Statistical analyses

Behavioral analyses did not include covariates. An analysis of variance (ANOVA) was used to assess effect of diagnosis on TOF. Linear regression was used to investigate the relationship between intrinsic interhemispheric SMN connectivity and TOF. We included an interaction term between SMN connectivity and diagnosis to test for group differences in this relationship while controlling for possible confounders, namely head motion as measured by mean framewise displacement (FD) and the head coil used during the scan. Due to the significant diagnostic difference in FSIQ score, imaging analyses were conducted with and without covarying for FSIQ and similar results were obtained. Therefore, the results reported do not include FSIQ as a covariate.

### 2.10 Data availability

Deidentified data included in this study is available upon request by any author listed on this manuscript.

## 3 Results

### 3.1 Effects of diagnosis on TOF, and SMN functional connectivity

#### 3.1.1 Behavioral assessment of total overflow (TOF)

**Fig. 3:**
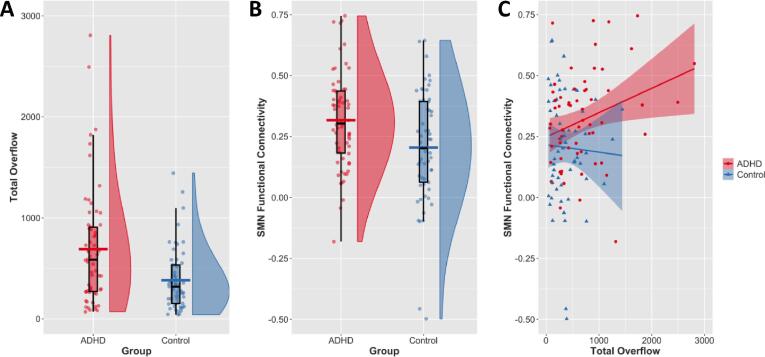
Association between SMN functional connectivity and total overflow by diagnosis.

There was a significant effect of diagnosis on TOF (*p* < 0.001; *d* = 0.671), such that children with ADHD showed greater TOF compared to TD children (ADHD$\overset{-}{x}$ = 690.33, standard deviation = 574.4; TD $\overset{-}{x}$ = 382.02, standard deviation = 303.1; see Fig. 3).

#### 3.1.2 SMN functional connectivity

As illustrated in Fig. 1**.**, right- and left-lateralized SMN components were localized to primary motor and primary somatosensory cortices of the respective hemispheres and to contralateral cerebellar motor regions. There was a significant effect of diagnosis on interhemispheric SMN functional connectivity such that children with ADHD exhibited increased interhemispheric connectivity compared to their TD peers (ADHD$\overset{-}{x}$ = 0.317, standard deviation = 0.197; TD $\overset{-}{x}$ = 0.205, standard deviation = 0.232; *p* = 0.023; *d* = 0.521; see Fig. 3B).

### 3.2 Association of SMN functional connectivity and diagnosis with TOF

#### 3.2.1 Diagnosis & SMN functional connectivity on TOF

We found a significant interaction between diagnosis and interhemispheric SMN functional connectivity on TOF (*t* = 2.348; *p* = 0.021). Children with ADHD who demonstrated stronger right-left interhemispheric SMN functional connectivity also demonstrated greater total overflow; a 0.1 increase in right-left SMN functional connectivity in the ADHD group resulted in a 93.2 increase in TOF compared with the TD group (Fig. 3). According to post hoc individual group regressions, this interaction was driven by the ADHD group showing a significant effect of SMN functional connectivity on TOF (*t* = 2.116; *p* = 0.039). Conversely, this effect was not observed in TD children (*t* = -0.444; *p* = 0.652).

## 4 Discussion

In this study, we applied group ICA to resting-state functional MRI to examine the neurobiological basis of ADHD-associated increases in mirror overflow. Consistent with our hypothesized diagnostic differences and published studies (MacNeil et al., 2011), we found that children with ADHD showed significantly greater mirror overflow compared to their TD peers. In addition, we found that children with ADHD showed significantly greater interhemispheric somatomotor network (SMN) functional connectivity compared to TD children. Critically, in children with ADHD, greater interhemispheric SMN functional connectivity was associated with greater mirror overflow, whereas we did not observe a similar relationship in TD children. Stronger functional connectivity between homologous somatomotor regions in children with ADHD may reflect impaired interhemispheric inhibition between these regions, which may diminish their ability to suppress overflow movements.

Our findings are consistent with the existing TMS literature investigating ipsilateral silent period (iSP) metrics related to neural inhibition in children with ADHD (Buchmann et al., 2003, Wu et al., 2012). Shorter iSP durations have been found in children with ADHD compared to TD peers, potentially reflecting an imbalance in corticomotor inhibitory and excitatory mechanisms, (Buchmann et al., 2013). Longer iSP latencies have also been observed in children with ADHD, and have been associated with greater ADHD symptom severity and increased motor overflow (Wu et al., 2012). Longer latencies could result from structural or functional abnormalities in motor regions or abnormalities in the myelination of transcallosal fibers between motor regions, leading to altered transcallosal inhibition (Garvey et al., 2005). These neurological findings of anomalous inhibition in children with ADHD complement our findings of increased mirror overflow exhibited in children with ADHD by offering support for the hypothesis that mirror overflow is a result of deficient inhibitory capacity between transcallosal motor regions.

Further support of deficient inhibition in children with ADHD is offered by Gilbert et al. (2011), who used TMS to exam short interval cortical inhibition (SICI), which is thought to be mediated by the inhibitory neurotransmitter GABA (γ-aminobutyric acid, Kujirai et al., 1993). Children with ADHD showed reduced SICI, which correlated with greater ADHD severity and poorer motor performance on the Physical and Neurological Examination of Subtle Signs, which includes a measure of motor overflow (Gilbert et al., 2011). In addition to reduced SICI, reduced GABA concentrations have been observed in children with ADHD compared to TD children (Edden et al., 2012). Both of these studies exemplifying deficient inhibition in children with ADHD support our hypothesis that an excessive mirror overflow in these children may be due to impaired inhibitory mechanisms between motor regions.

Our findings are also consistent with existing structural MRI examinations of the corpus callosum (CC) in ADHD. McNally et al., found that girls with ADHD showed a relationship between measures of response control on a Go/No-Go task and the circumference of the isthmus (McNally et al., 2010). Given that the isthmus projects to primary motor and primary somatosensory cortices, this finding suggests that poor performance on a Go/No-Go task in girls with ADHD could be due to structural abnormalities in these regions. Luders et al. found that male adolescents with ADHD showed thinner CCs than their TD peers in anterior regions and, particularly, in posterior regions, whose fibers project to the parietal cortex, where the post-central gyrus is located (Luders et al., 2009). In a sample of male adults, Luders et al. observed a negative relationship between callosal thickness and ADHD symptomology, such that thinner callosal thickness was associated with greater inattention and hyperactivity (Luders et al., 2016). Thinner CCs would suggest the presence of fewer fibers or less myelination of fibers in the CC through which transcallosal connections can be formed. Given the positive relationship between fiber microstructure in the CC and interhemispheric inhibition between motor regions (Koerte et al., 2009), a decrease in the number of and myelination of fibers in the CC could lead to deficits in the transcallosal inhibition contributing to motor overflow.

It is possible that both inhibitory and excitatory signaling would be affected by abnormalities in the CC. However, a study using TMS to examine inhibitory and excitatory signaling found that while children with ADHD show slightly higher intra-cortical facilitation compared to their TD peers, they show a more robust decrease in intra-cortical inhibition (Wu et al., 2012). Additionally, reductions in intra-cortical and transcallosal inhibition in children with ADHD was associated with more severe ADHD symptoms and motor impairment (Gilbert et al., 2011, Wu et al., 2012). These findings suggest that disrupted inhibitory, more so than excitatory, mechanisms may underlie our observations of increased inter cortical SMN network connectivity and mirror overflow in children with ADHD.

Together, these behavioral and neural findings give way to discussions about their practical and clinical significance. Atypical motor development, including the presence of excessive motor overflow, can negatively impact a child’s functioning in school and in social settings. For example, extraneous movements could hinder one’s performance in playing instruments or participating in athletics. Though motor overflow typically diminishes in adolescence as the motor system matures, findings from the current and previous literature can be used to create therapies for more quickly ameliorating excessive motor overflow (Szatmari & Taylor, 1984).

We should note a few limitations of the current study. First, our overall sample size was modest and included a small number of girls which limited our ability to examine effects of sex in our statistical models and generalize findings to girls with ADHD. Second, while strong correlations between resting state functional connectivity and mirror overflow suggests that interhemispheric somatomotor functional connectivity is important for suppression of this behavior, we can only speculate about the order of events that results in this brain-behavior correlation. Studying fMRI data alongside other imaging data, such as EEG or TMS, would help to understand the functional connectivity of networks directly underlying persistence of mirror overflow in children with ADHD.

Therefore, future studies would benefit from the inclusion of electrophysiological measures of interhemispheric inhibition from TMS to examine interhemispheric inhibition of transcallosal networks that accompanies mirror overflow in children with ADHD. Furthermore, inclusion of diffusion tensor imaging (DTI) data would be advantageous for understanding whether structural abnormalities in interhemispheric connectivity are also associated with mirror overflow, in parallel to the current findings of increased interhemispheric SMN functional connectivity in ADHD. Lastly, an analysis of functional connectivity data during a finger sequencing task would allow for insight on the extent of interhemispheric functional connectivity between SMNs while motor overflow is occurring.

## 5 Conclusions

In summary, the present study investigated associations among diagnosis, interhemispheric SMN functional connectivity, and total overflow in children with and without ADHD. Children with ADHD demonstrated greater total overflow and interhemispheric SMN functional connectivity compared to their TD peers, as well as a positive relationship between SMN functional connectivity and TOF. The ADHD-associated increase in interhemispheric SMN connectivity we observed is consistent with the theory that interhemispheric cortical inhibitory mechanisms are disrupted in ADHD, leading to an impaired ability to suppress motor overflow movements. Future studies should continue to build on this work investigating the link between the neurobiology underlying unwanted movements and the mechanisms critical for impaired behavioral inhibitory capacity more generally in ADHD.

## CRediT authorship contribution statement

**C. Chen:** Conceptualization, Data curation, Formal analysis, Investigation, Visualization, Writing - original draft, Writing - review & editing. **D. Lidstone:** Data curation, Formal analysis, Software, Visualization, Writing - original draft, Writing - review & editing. **D. Crocetti:** Investigation, Resources, Project administration, Writing - review & editing. **S.H. Mostofsky:** Conceptualization, Funding acquisition, Supervision, Writing - review & editing. **M.B. Nebel:** Conceptualization, Data curation, Formal analysis, Methodology, Project administration, Software, Supervision, Writing - original draft, Writing - review & editing.

## Declaration of Competing Interest

The authors declare that they have no known competing financial interests or personal relationships that could have appeared to influence the work reported in this paper.
